# Natural Multifunctional Silk Microcarriers for Noise‐Induced Hearing Loss Therapy

**DOI:** 10.1002/advs.202305215

**Published:** 2023-11-20

**Authors:** Hui Zhang, Hong Chen, Ling Lu, Huan Wang, Yuanjin Zhao, Renjie Chai

**Affiliations:** ^1^ State Key Laboratory of Digital Medical Engineering, Department of Otolaryngology Head and Neck Surgery, Zhongda Hospital, School of Life Sciences and Technology, Advanced Institute for Life and Health, Jiangsu Province High‐Tech Key Laboratory for Bio‐Medical Research Southeast University Nanjing 210096 China; ^2^ Department of Rheumatology and Immunology, Nanjing Drum Tower Hospital, School of Biological Science and Medical Engineering Southeast University Nanjing 210096 China; ^3^ Co‐Innovation Center of Neuroregeneration Nantong University Nantong 226001 China; ^4^ The Eighth Affiliated Hospital Sun Yat‐sen University Shenzhen 518033 China; ^5^ Department of Otolaryngology Head and Neck Surgery, Sichuan Provincial People's Hospital University of Electronic Science and Technology of China Chengdu 610072 China; ^6^ School of Life Science Beijing Institute of Technology Beijing 100081 China; ^7^ Guangdong Key Laboratory for Biomedical Measurements and Ultrasound Imaging, Department of Biomedical Engineering Shenzhen University Shenzhen 518060 China

**Keywords:** drug delivery, hearing loss, inverse opal, polydopamine, silk fibroin

## Abstract

Noise‐induced hearing loss (NIHL) is a common outcome of excessive reactive oxygen species in the cochlea, and the targeted delivery of antioxidants to the inner ear is a potential therapeutic strategy. In this paper, a novel natural biomaterials‐derived multifunctional delivery system using silk fibroin‐polydopamine (PDA)‐composited inverse opal microcarriers (PDA@SFMCs) is presented for inner ear drug delivery and NIHL therapy. Due to their large specific surface area and interpenetrating nanochannels, PDA@SFMCs can rapidly load active biomolecules making them a convenient medium for the storage and delivery of such molecules. In addition, surface modification of PDA enables the microcarriers to remain in the round window niche, thus facilitating the precise local and directed delivery of loaded drugs. Based on these features, it is demonstrated here that *n*‐acetylcysteine‐loaded silk microcarriers have satisfactory antioxidant properties on cells and can successfully prevent NIHL in guinea pigs. These results indicate that the natural multifunctional silk microcarriers are promising agents for local inner ear drug delivery in the clinic.

## Graphical abstract

A type of multifunctional delivery system with silk fibroin‐polydopamine composited inverse opal microcarriers (PDA@SFMCs) is presented for inner ear drug delivery and noise‐induced hearing loss therapy. PDA@SFMCs can rapidly load actives, achieving convenient usage and storage. Besides, PDA surface modification enables the long‐term retention of microcarriers on the round window niche, facilitating local and directional inner ear drug delivery.

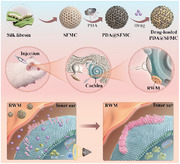

## 1 Introduction

Noise‐induced hearing loss (NIHL) acquired in occupational settings is one of the most common causes of hearing impairment, especially in industrialized countries.^[^ ^1^ ^]^ Under noise exposure, excess reactive oxygen species (ROS) in the cochlea are produced, and endogenous antioxidant systems are often insufficient at ROS scavenging.^[^ ^2^ ^]^ In such cases, exogenous antioxidants can play a positive role in maintaining homeostasis.^[^ ^3^ ^]^ However, the blood‐labyrinth barrier prevents the effective accumulation of therapeutic agents that are administered by traditional methods such as systemic administration and intravenous injection.^[^ ^4^ ^]^ In comparison, local administration through the round window membrane (RWM) has emerged as a preferred strategy, and thus various biomaterial‐derived vehicles have been developed for local inner ear delivery, including nanoparticles, bulk hydrogels, microbumbs, and microparticles.^[^ ^5^ ^]^ Despite ongoing progress in the field, most of these carriers have simple structures, poor long‐term storage of the loaded drugs, and an inability to remain at the target position, thus resulting in unsatisfactory outcomes. In addition, these carriers are mainly derived from synthetic polymers, which usually lead to poor biocompatibility and insufficient biodegradation. Therefore, new vehicles for NIHL treatment are still needed.

**Figure 1:**
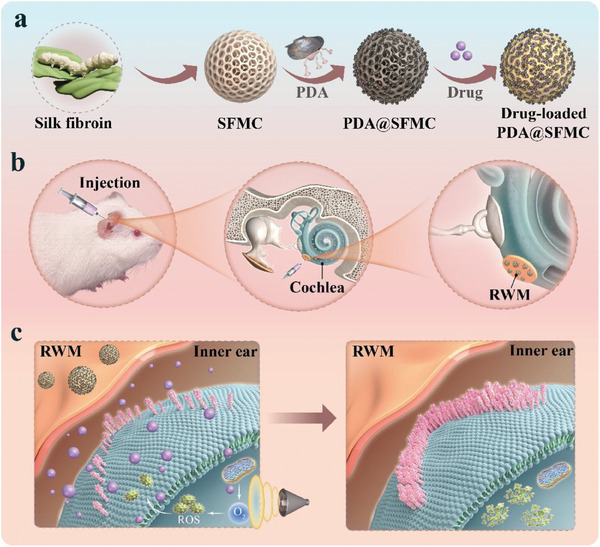
Schematic diagram of natural multifunctional silk microcarriers for NIHL therapy. a) Preparation of drug‐loaded PDA@SFMCs. b) Schematic of the injection procedure. c) Scheme of the drug delivery process crossing the RWM into the inner ear.

In this work, we present a novel natural multifunctional silk fibroin microcarrier with adhesive capability that is ready‐to‐use for locally sustained drug delivery and hearing loss therapy, as shown in **Figure** **1**. Silk fibroin, which comes from silkworm cocoons, has been extensively applied for various biomedical applications, especially drug carrier construction.^[^ ^6^ ^]^ It has many useful properties, including excellent biocompatibility, low toxicity, no immunogenicity, superior mechanical performance, and adjustable biodegradability.^[^ ^7^ ^]^ Polydopamine (PDA) is a mussel‐derived material with excellent wet adhesion and free radical‐scavenging capability, which are due to its catechol and primary amine groups, thus making it a suitable candidate for enhancing the adhesion of biomaterials.^[^ ^5^, ^8^ ^]^ Inverse opal is a material with spatially ordered porous structures generally obtained by negative replication of a colloidal crystal template.^[^ ^9^ ^]^ Studies have confirmed that such unique porous structures endow inverse opal with a high specific surface area and connected nanochannels, and these are conducive to the rapid loading and sustained release of various substances.^[^ ^10^ ^]^ Thus we had the idea to integrate these elements and construct a novel silk fibroin‐PDA composite inverse opal microcarrier in the hopes that it would have better adhesion and drug loading properties compared to currently available materials.

We constructed the desired composite silk fibroin microcarriers with PDA modification and inverse opal structure (PDA@SFMCs) through a negative replication stratagem and tested it for inner ear drug delivery and NIHL therapy. The generated PDA@SFMCs exhibited satisfactory biocompatibility, slow degradation, richly porous nanostructures, and interpenetrating nanochannels. Benefitting from such unique nanostructures, PDA@SFMCs could rapidly absorb therapeutic molecules prior to use, suggesting that the material can serve as a ready‐to‐use drug vehicle with long‐term storage capability. Additionally, the PDA modification allowed the microcarriers to be locally administered through the round window niche, allowing for precise local and targeted delivery of the loaded drugs. When loaded with the antioxidant *N*‐acetylcysteine (NAC), the silk microcarriers showed antioxidant characteristics in cells. In vivo experiments confirmed that NAC‐loaded silk microcarriers could effectively prevent hearing loss, reduce hair cell deficiency, and alleviate synaptic damage in noise‐exposed guinea pigs. These results suggest that our natural silk microcarriers are a promising tool for local inner ear drug delivery and hearing loss therapy.

## 2 Results and Discussion

**Figure 2:**
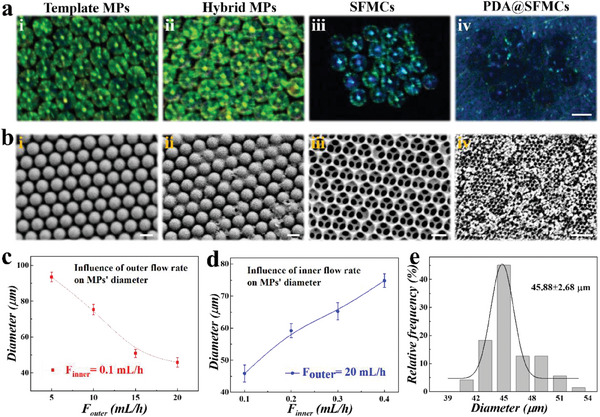
Characterization of the resultant MPs. a) Bright‐field images of i) template MPs, ii) hybrid MPs, iii) SFMCs, and iv) PDA@SFMCs. b) SEM images of the surfaces of i) template MPs, ii) hybrid MPs, iii) SFMCs, and iv) PDA@SFMCs. c) Influence of the outer flow rate and d) the inner flow rate on the MP template diameter. e) Size distribution of template MPs with an inner flow velocity of 0.1 mL h^−1^ and an outer flow velocity of 20 mL h^−1^. Scale bars are 50 µm in (a), 200 nm in (b‐i–iii), and 1.2 µm in (b‐iv).

In a typical experiment, silica microparticles (MPs) were generated through droplet microfluidics to provide a replication template for inverse opal construction. The synthesized silica nanoparticles were dispersed in ultra‐pure water and collected into an injector, followed by the generation of droplets via a microfluidic glass device and two peristaltic pumps. During the process of drying and calcination, silica nanoparticles assembled into the template MPs with ordered close‐packing structures (**Figure** **2**a‐i), which provided abundant nanovoids for substance infiltration. For the fabrication of silk‐derived inverse opal particles, the methylacrylylated silk fibroin solution containing a photoinitiator was used to fill the nanopores of the template MPs, and then ultraviolet light was applied to gel the silk fibroin. The removal of redundant hydrogel on the outside of the template MPs resulted in silk fibroin hybrid MPs (Figure 2a‐ii), and SFMCs were obtained after hydrofluoric acid corrosion (Figure 2a‐iii). To endow the microspheres with ideal adhesion, SFMCs were modified with PDA, and the generated particles were referred to as PDA@SFMCs, as shown in Figure 2a‐iv. The microstructures of the generated microparticles, including the template MPs, silk hybrid MPs, SFMCs, and PDA@SFMCs, were analyzed by scanning electron microscopy (SEM), as shown in Figure 2b. It was found that the template MPs showed hexagonally close‐packed structures both on the surface and inside (Figure 2b‐i and Figure S1a, Supporting Information). Due to the nanovoids between the silica nanoparticles, the pre‐gel successfully permeated the MPs and ultimately formed inverse opal particles with highly uniform and porous structures that contributed to the rapid drug loading and sustained release (Figure 2b‐ii and Figure S1b, Supporting Information). Especially important, by varying the flow rates of the outer and inner fluids in the microfluidics device the size of template MPs could be precisely controlled. As shown in Figure 2c,d, the diameter of the MPs decreased by increasing the outer flow rate and increased by increasing the inner flow rate. For the most convenient injection, 0.1 and 20 mL h^−1^ were selected as the final inner flow rate and outer flow rate, respectively, and the resultant MPs had a uniform diameter of around 45.88 µm (Figure 2e).

We next tested the swelling behavior and biocompatibility of the resultant PDA@SFMCs. Because we sought to use this material in the inner ear microenvironment, artificial perilymph solution (APL) was prepared according to a previous study for swelling and degradability testing.^[^ ^11^ ^]^ As shown in Figure S2, Supporting Information, the lyophilized PDA@SFMCs reached swelling equilibrium after a 4 h immersion in APL at around 637.92 ± 22.19%, and this was beneficial for rapid drug loading. In addition, the cytotoxicity of PDA@SFMCs to House Ear Institute‐Organ of Corti 1 (HEI‐OC1) cells was determined using a live/dead staining kit and a cell counting kit (CCK‐8). Figure S3a–c, Supporting Information shows that HEI‐OC1 cells co‐cultured with PDA@SFMCs had a morphology similar to that in normal medium without PDA@SFMCs (which was considered the control group) at days 1, 2, and 3, and there were almost no dead cells. Also, the CCK‐8 test showed the same result, as shown in Figure S3d, Supporting Information. These results indicated the satisfactory biocompatibility of the prepared PDA@SFMCs for their further in vivo use.

**Figure 3:**
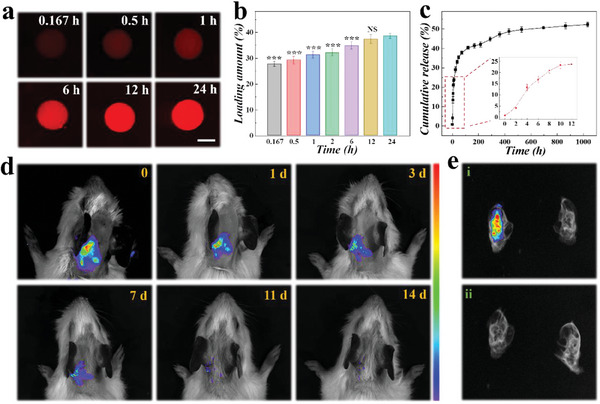
Drug loading and release behaviors of PDA@SFMCs. a) Confocal scanning photographs of RHB‐loaded PDA@SFMCs with different immersion times. b) The drug‐loading amount in PDA@SFMCs over 24 h. c) Release kinetics of RHB from PDA@SFMCs in APL. d) IVIS images of guinea pigs with Cy7‐loaded PDA@SFMCs over the course of 14 days. e) in vivo imaging systemIVIS image of the isolated cochlea 12 h after injection with i) Cy7‐loaded PDA@SFMCs or ii) Cy7 alone. Scale bar is 25 µm.

To determine the drug loading and release behaviors, we chose Rhodamine B (RHB) as the model drug instead of NAC for in vitro drug release evaluation because of its intrinsic fluorescence and its similar molecular weight to NAC. About 27.73 ± 1.03% of the RHB could be loaded on PDA@SFMCs after a 10‐min immersion, and 37.38 ± 1.76% of the RHB was loaded after 12 h, at which time the loading capacity of PDA@SFMCs reached saturation (**Figure** **3**a,b). This result demonstrated the rapid loading capability of PDA@SFMCs, which is likely due to the interconnected nanochannels and high specific surface area of the material. Additionally, it was observed from the confocal laser scanning images that RHB with red fluorescence was evenly distributed in the entire PDA@SFMC, demonstrating the complete loading of drugs after a 12‐h immersion (Figure S4, Supporting Information). Next, the in vitro release kinetics of PDA@SFMCs were investigated through the standard curve method, and the standard curve of RHB in APL was first recorded as shown in Figure S5a, Supporting Information. The cumulative release curve of RHB released from PDA@SFMCs was then calculated by measuring the RHB absorbance in APL. As shown in Figure 3c, the loaded RHB had a slow and sustained release process, which is significant for the long‐term delivery of small molecular weight drugs with PDA@SFMCs.

We then investigated the entry of drugs into the cochlea through the RWM after the drug‐loaded PDA@SFMCs had been injected into the RWM niche. As shown in Figure 3d, the near‐infrared fluorescent dye Cyanine 7 (Cy7) was loaded into PDA@SFMCs, and an in vivo imaging system (IVIS) was used for 14 days of observation. Notably, as a commonly used near‐infrared fluorescent dye, Cy7 could generate signals in deep tissues and was used to simulate drug molecules for tracing by IVIS. It was found that the injected Cy7‐loaded PDA@SFMCs’ fluorescence signal lasted for over 14 days and showed a slow rate of decrease (Figure S5b, Supporting Information). In addition, after a 12‐h implantation of PDA@SFMCs, apparent fluorescence signals were observed in the inner ear of the Cy7‐loaded PDA@SFMC group, showing that the drug could passively penetrate into the inner ear through the RWM (Figure 3e). In contrast, little fluorescence was found in the inner ear of the control group (directly injected with Cy7). These results demonstrated the significant adhesion of PDA@SFMCs, which increased the residence time of drug‐loaded microspheres at the RWM and improved the loaded drug's bioavailability.

**Figure 4:**
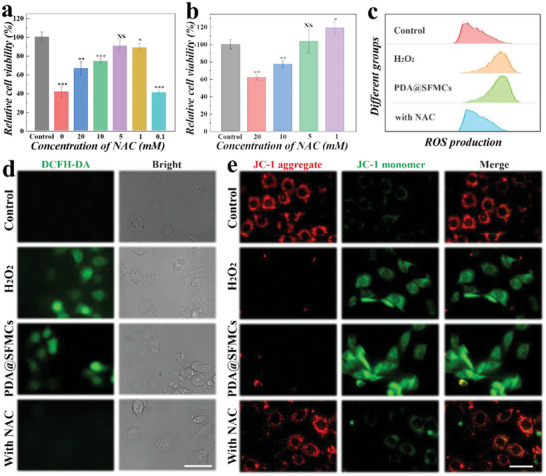
The antioxidant capacity of NAC‐loaded PDA@SFMCs on HEI‐OC1 cells. a) The relative cell viability of H_2_O_2_‐damaged and NAC‐pretreated HEI‐OC1 cells at various NAC concentrations. b) The relative viability of cells that were exposed to various concentrations of NAC. c) ROS production in cells as measured by flow cytometry. d) Photographs of DCFH‐DA staining. e) JC‐1 staining images. Scale bars are 50 µm.

To explore the antioxidant capacity of NAC‐loaded PDA@SFMCs, we constructed an HEI‐OC1 cell model injured by hydrogen peroxide (H_2_O_2_). First, the optimal concentration and treatment time for H_2_O_2_‐induced HEI‐OC1 cell injury were determined. We co‐cultured cells with a gradient of H_2_O_2_ (0, 25, 50, 75, 100, 200 µm) for 4, 8, and 12 h, respectively, and cell viability was determined by the CCK‐8 method. According to the results shown in Figure S6, Supporting Information, we chose 200 µm and 8 h as the optimized concentration and treatment time for the subsequent experimental conditions. Under such conditions, the HEI‐OC1 cells were obviously damaged, and the cell viability was 48.3% ± 4.65%. After that, the protective concentrations of NAC were determined. NAC at a concentration of 0.1, 1, 5, 10, and 20 mm was used to pretreat HEI‐OC1 cells for 1 h, and then the cells were damaged using the H_2_O_2_ treatment conditions described above. When the concentration of NAC was too low, it could not protect the cells from H_2_O_2_ damage, and when the concentration was too high it had toxic side effects (**Figure** **4**a). Furthermore, it was found that a high dose of NAC negatively impacted cell viability (Figure 4b). As a result, a NAC concentration of 1 mm was selected to protect cells from H_2_O_2_ damage.

Generally, H_2_O_2_ can cause excessive production of ROS, thereby triggering mitochondrial dysfunction leading to cell damage. In this study, the ROS level within HEI‐OC1 cells was measured using the fluorescent probe 2′,7′‐dichlorofluorescein diacetate (DCFH‐DA), the fluorescent signal of which increases with the ROS level. As shown in Figure 4d, the green fluorescence signal of cells treated with H_2_O_2_ was elevated in comparison to that of the control group, demonstrating that H_2_O_2_ induced increased ROS generation. Compared to the group treated with pure PDA@SFMCs, the fluorescence signal of the cells in the NAC‐loaded PDA@SFMCs group was weak, which demonstrated the effective reduction of the intracellular H_2_O_2_‐induced ROS accumulation triggered by NAC released from the PDA@SFMCs. The flow cytometry results were comparable to those of fluorescence detection, as indicated in Figure 4c. In addition, we detected the cells' mitochondrial membrane potential (MMP) using JC‐1. In normal cells, high MMP leads to the formation of JC‐1 aggregates in the mitochondrial matrix that emit red fluorescence, while low MMP makes JC‐1 appear as a monomer and emit green fluorescence. Therefore, we evaluated MMP changes by examining the JC‐1 fluorescence signal transition as a reflection of the level of cell apoptosis. As shown in Figure 4e, the red fluorescence of the cells in the H_2_O_2_ treatment group and the pure PDA@SFMCs group was less intense than that in the control group, and instead intense green fluorescence was observed. At the same time, HEI‐OC1 cells in the NAC‐loaded PDA@SFMCs treatment group emitted a strong red fluorescent signal, which indicated an increase in JC‐1 aggregates. These results indicated the effectiveness of NAC released from microspheres in maintaining mitochondrial function and cell viability.

**Figure 5:**
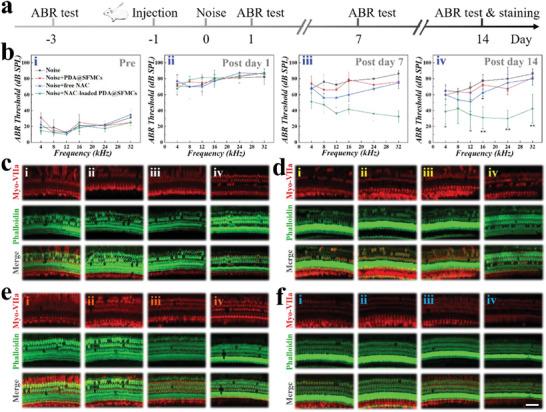
In vivo evaluation of NAC‐loaded PDA@SFMCs on hearing loss therapy. a) Timeline of the animal experiments. b) ABR thresholds in each group i) prior to noise exposure and at ii) 1, iii) 7, and iv) 14 days afterward. c–f) Immunofluorescence staining images of OHCs in the i) apical turn, ii) third turn, iii) second turn, and iv) basal turn of c) the control group, d) PDA@SFMC‐treated group, e) free NAC‐treated group, and f) NAC‐loaded PDA@SFMC‐treated group. Hair cells were stained with Myosin VIIa (red) and Phalloidin (green). Scale bar is 50 µm.

To assess the impact of NAC‐loaded PDA@SFMCs in hearing loss therapy, we constructed a guinea pig noise injury model (**Figure** **5**a). Before the operation, the normal hearing levels were determined through auditory brainstem response (ABR) tests (Figure 5b‐i). One day before noise exposure, PDA@SFMCs, free NAC, and NAC‐loaded PDA@SFMCs were injected through the auditory bulb. The guinea pigs were then exposed to 119 dB white noise for 4 h resulting in a permanent shift of the ABR thresholds (Figure 5b‐ii). The statistical analysis showed that 14 days after noise exposure the NAC‐loaded PDA@SFMCs group had the most obvious hearing recovery, which was statistically different from the direct injection of NAC (Figure 5b‐iv). This result indicated that NAC‐loaded PDA@SFMCs successfully delivered drugs into the inner ear, and due to their excellent adhesion, they could release drugs slowly and continuously in situ at the RWM, thus reducing drug loss and improving efficacy. Subsequently, in order to explore the damage to guinea pig outer hair cells (OHCs) after noise exposure the morphology of OHCs was observed using immunofluorescence staining and confocal microscopy, with myosin‐VIIa (myo‐VIIa) and Phalloidin being used to mark hair cells. As shown in Figure 5c–f and Figure S7, Supporting Information, excessive noise exposure caused severe loss of OHCs, while the NAC treatment group improved the survival rate of OHCs to some extent. Importantly, the loss rate of OHCs in the NAC‐loaded PDA@SFMCs treatment group was reduced. These results confirmed that NAC‐loaded PDA@SFMCs could promote the survival of hair cells.

**Figure 6:**
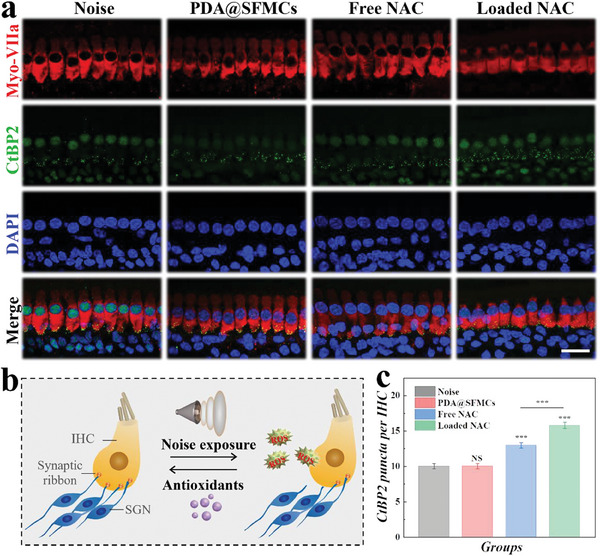
CtBP2 expression in IHCs. a) Immunofluorescence images of CtBP2 expression in IHCs. IHCs were labeled with red fluorescence, synaptic ribbons were labeled with green fluorescence, and the nuclei were stained with DAPI (blue). b) Scheme showing the influence of noise exposure and antioxidant (NAC) treatment on IHCs. SGN is the spiral ganglion neuron. c) The CtBP2 puncta per IHC in the different groups. Scale bar is 20 µm.

CtBP2‐positive areas on inner hair cell (IHC) synapses make it possible to quantify the damaged synaptic ribbons in the IHC region caused by noise overexposure (**Figure** **6**). The degree of synaptic damage in the NAC group was diminished but remained less than that of the NAC‐loaded PDA@SFMC treatment group, indicating that the amount of drug entering the inner ear through the RWM increased. As mentioned above, these properties could be attributed to the strong adhesion of NAC‐loaded PDA@SFMCs to the RWM, which allowed NAC to avoid being cleared through the Eustachian tube and instead be released into the inner ear.

## 3 Conclusion

In summary, we have developed a silk microcarrier with PDA modification and inverse opal structures for local drug delivery and hearing loss therapy. The resultant PDA@SFMCs were shown to have richly porous nanostructures and interconnected nanochannels that were inherited from the MP templates and contributed to the rapid loading and continuous release of drugs. We confirmed that the silk microcarriers had good biocompatibility as well as satisfactory delivery of antioxidants in HEI‐OC1 cells. Due to the PDA modification, the resultant microcarriers could remain localized at the RWM for a long time, which was beneficial for the precise local and targeted delivery of loaded drugs. The in vivo study results revealed that the prepared NAC‐loaded PDA@SFMCs could partly restore hearing levels, reduce OHC loss, and reduce synaptic ribbon damage in guinea pigs after noise exposure. These results validated that PDA@SFMC is a promising innovation for local inner ear drug delivery for hearing loss therapy.

## 4 Experimental Section

### Preparation of PDA@SFMCs

A silica nanoparticle solution was used as the dispersed phase of the microfluidic system, and methyl silicone oil was used as the continuous phase. The dispersed phase formed uniform droplets in the microfluidic device due to fluid shear force, and colloidal crystal microsphere templates were obtained after drying and calcination. Afterward, the photocurable silk fibroin pre‐gel solution at a concentration of 15 wt% was prepared, and the colloidal crystal microspheres were incubated in it for 12 h, followed by ultraviolet light irradiation for 30 s. The template microspheres were removed by 4 wt% hydrofluoric acid to obtain SFMCs . The SFMCs were placed in tris‐HCl solution (10 mm, pH 8.5) with 2 mg mL^−1^ dopamine. After stirring for 12 h and washing, PDA@SFMCs were obtained.

### Swelling Test

A total of 1 mL of APL was added to a 2 mL EP tube containing 6 mg of freeze‐dried PDA@SFMCs. The APL consisted of 10 mm D‐glucose, 150 mm NaCl, 4 mm KCl, 1.5 mm CaCl_2_, 1 mm MgCl_2_, 2 mm NaH_2_PO_4_, and 8 mm Na_2_HPO_4_, which was similar to the liquid within the cochlea. The EP tube was placed on a shaker with a rotational speed of 100 rpm min^−1^ and a constant temperature of 37 °C. The supernatant was removed at specific times, and the PDA@SFMCs were collected for weight determination. The swelling rate formula was:

(1) $$\mathrm{Swelling}\;\mathrm{rate}\;=\frac{\mathrm{Weigh}t_{\mathrm{wet}}-\mathrm{Weigh}t_{\mathrm{dry}}}{\mathrm{Weigh}t_{\mathrm{dry}}}\;\times \;100$$

### Drug Loading and Release

First, 1 mg mL^−1^ of RHB was prepared as a small molecule mimic drug, and the prepared PDA@SFMCs were soaked in the RHB solution. The PDA@SFMCs were removed at different time points for observation with a confocal microscope. For the drug loading assay the supernatant was siphoned off at specific time points, and the absorbance of the supernatant was measured so as to calculate the encapsulation efficiency. Next, the release kinetics of PDA@SFMCs were determined. The RHB‐loaded PDA@SFMCs were soaked in the pre‐configured APL, and 100 µL supernatant was removed at each specific time point to measure the absorbance, and an equivalent volume of fresh buffer was added to maintain the total volume.

### Biocompatibility Test

The HEI‐OC1 cell line was selected to test the prepared PDA@SFMCs' cytotoxicity. The PDA@SFMCs were co‐cultured with 5 × 10^4^ HEI‐OCI cells in a 48‐well plate. The live/dead staining kit was then used to stain the cells at 1, 2, and 3 days, and the CCK‐8 agent (at a 1:9 ratio) was used to evaluate cell proliferation. After incubation for 2 h, 100 µL of the supernatant was taken to measure the absorbance.

### Drug Delivery across the RWM

Cy7 alone or Cy7‐loaded PDA@SFMCs were injected into the round window niche of guinea pigs, and fluorescence signals were detected by IVIS spectroscopy. Images were collected on days 0, 1, 3, 5, 7, 11, and 14. Additionally, to confirm that small molecule drugs released from PDA@SFMCs could enter the inner ear, the guinea pig cochlea was collected 12 h after injection, and IVIS images were obtained again.

### ROS Level and MMP Evaluation

In a 48‐well plate, 5 × 10^4^ HEI‐OC1 cells in each well were incubated with different concentrations of H_2_O_2_ solution diluted in culture medium without fetal bovine serum for 4, 8, or 12 h. The CCK‐8 kit was then used to measure the cell viability of each group to determine the most suitable treatment time and concentration of H_2_O_2_. In order to evaluate the impact of NAC on HEI‐OC1 cells, the cells were directly treated with different doses of NAC for 1 h and the cell viability was detected. After that, the HEI‐OC1 cells were pretreated with NAC solution at an optimal concentration for 1 h, then treated with H_2_O_2_ at a concentration of 200 µm for 8 h, and measured the cell viability using a CCK‐8 kit. A ROS kit was used to measure the ROS level in HEI‐OC1 cells. Immediately afterward the DCFH‐DA probe was incubated for 20 min and rinsed three times, and images were acquired using a Leica microscope. In addition, flow cytometry was used to quantify the generation of ROS. The JC‐1 probe was used to stain cells for 20 min, the cells were rinsed with matching buffer solution, and images were acquired immediately using a Leica microscope.

### NIHL Model in Guinea Pigs

The Animal Ethics Committee of Zhongda Hospital, Southeast University School of Medicine, approved all animal experimental procedures (20220301040). The hearing ability of guinea pigs was tested by ABR, and white male guinea pigs with normal hearing were selected and randomly divided into four groups, including the Noise group, Noise+PDA@SFMCs group, Noise+NAC, group, and Noise+NAC‐loaded PDA@SFMCs group. After fasting for 12 h, the guinea pigs were anesthetized with isoflurane, and then 2 wt% lidocaine was given behind the ears for local anesthesia. In order to avoid the adverse effect of the electric drill on the hearing of the guinea pigs, a scalpel was used to drill holes to expose the round window niche. For the animals in the Noise+NAC group, NAC was directly injected through the auditory bulb, while for the animals in the Noise+PDA@SFMCs group and the Noise+NAC‐loaded PDA@SFMCs group, the non‐NAC‐loaded/NAC‐loaded PDA@SFMCs were injected into the RWM with a syringe. After the guinea pigs woke up from anesthesia they were put in a specially constructed wire cage, and white noise with a sound pressure level of 119 dB was provided for 4 h. ABR tests were carried out on days 1, 7, and 14 following noise exposure.

### ABR Test

Guinea pigs were deeply anesthetized and placed in the Tucker‐Davis Technologies System workstation compartment and tested at 4, 8, 12, 16, 24, and 32 kHz. Every frequency was checked from 90 dB, and all recorded data were collated and analyzed using Origin software.

### Immunofluorescent Staining

At 14 days after noise exposure, the guinea pigs were sacrificed, the temporal bone was collected, and the cochlea was fully exposed and immediately immersed in 4 wt% paraformaldehyde and fixed overnight at 4 °C. The cochlea was washed in PBS, and ethylene diamine tetraacetic acid at a concentration of 0.5 m was introduced and changed daily for 1 week. The cochlea was then washed in PBS, and the cochlea slices were processed under a microscope. For cochlear hair cells, myosinVIIa and Phalloidin were used for staining and observation, and for inner ear hair cell synaptic structures CtBP2 was used as a presynaptic marker for staining and observation. For staining, the samples were fixed at room temperature for 1 h, permeabilized for 15 min, and incubated with the corresponding primary antibody dilution overnight at 4 °C. The next day, the samples were washed with PBS and 0.1 vol% Triton X‐100 (PBST) and incubated with the secondary antibody at room temperature for around 1 h. Finally, the samples were rinsed with PBST, treated with an anti‐fluorescent quencher (DAKO), and sealed with nail polish for subsequent observation.

### Statistical Analysis

The results were analyzed using Origin software and are presented as the mean ± SEM. Student's *t*‐test or one‐way ANOVA followed by the Tukey post‐hoc test was performed to evaluate whether differences were of statistical significance. Statistical significance was defined as NS: no significant, *p < 0.05, **p < 0.01, and ***p < 0.001.

## Conflict of Interest

The authors declare no conflict of interest.

## Author Contributions

Y.Z. and R.C. conceived the idea. H.Z. designed and conducted the experiments and data analysis. H.C., L.L., and H.W. examined the data. H.Z., H.W., and Y.Z. wrote and revised the manuscript.

## Supporting information

Supporting Information

## Data Availability

The data that support the findings of this study are available from the corresponding author upon reasonable request.
