# Supplementary material for: Natural Multifunctional Silk Microcarriers for Noise‐Induced Hearing Loss Therapy
Source: Adv Sci (Weinh). 2023 Nov 20;11(1):2305215. doi: 10.1002/advs.202305215 (PMC10767431; doi:10.1002/advs.202305215)
Supplement: Supplementary file 1 — Supporting Information [file ADVS-11-2305215-s001.pdf]

ADVANCED SCIENCE
Open Access

Supporting Information

for *Adv. Sci.*, DOI 10.1002/advs.202305215

Natural Multifunctional Silk Microcarriers for Noise-Induced Hearing Loss Therapy

Hui Zhang, Hong Chen, Ling Lu, Huan Wang, Yuanjin Zhao* and Renjie Chai**

Supporting Information

Natural multifunctional silk microcarriers for noise-induced hearing loss therapy

Hui Zhang, Hong Chen, Ling Lu, Huan Wang, Yuanjin Zhao*, Renjie Chai**

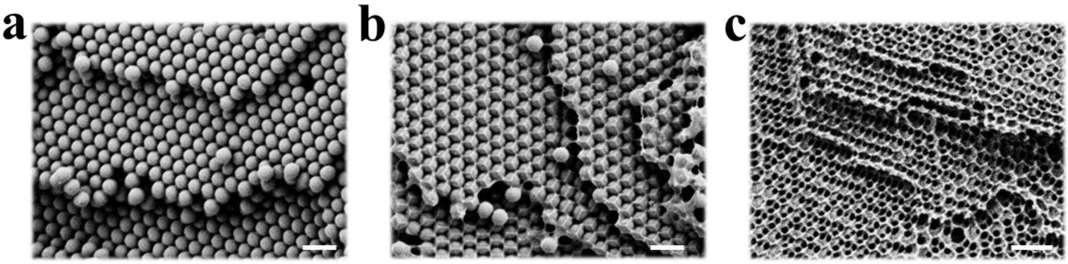

Figure S1. SEM images of interior structures of template MPs (a), hybrid MPs (b) and SFMCs (c). Scale bars are 500 μm.

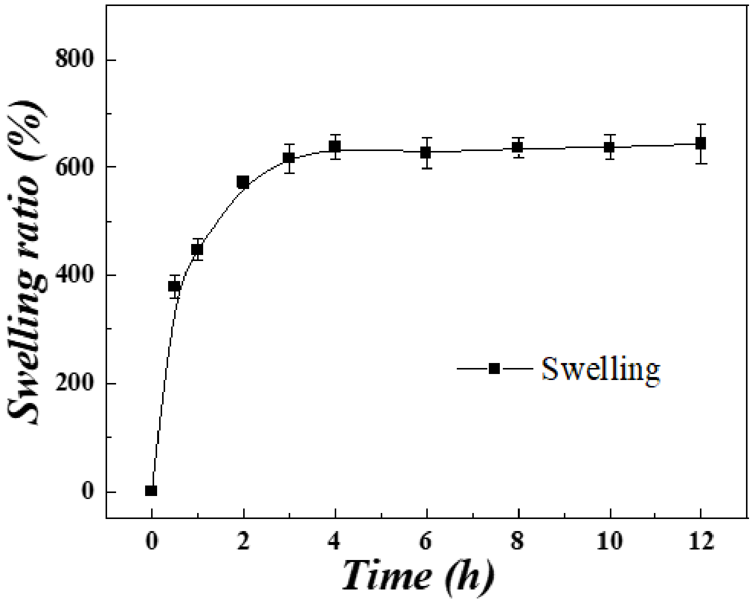

Figure S2. Swelling property of PDA@ SFMCs in APL.

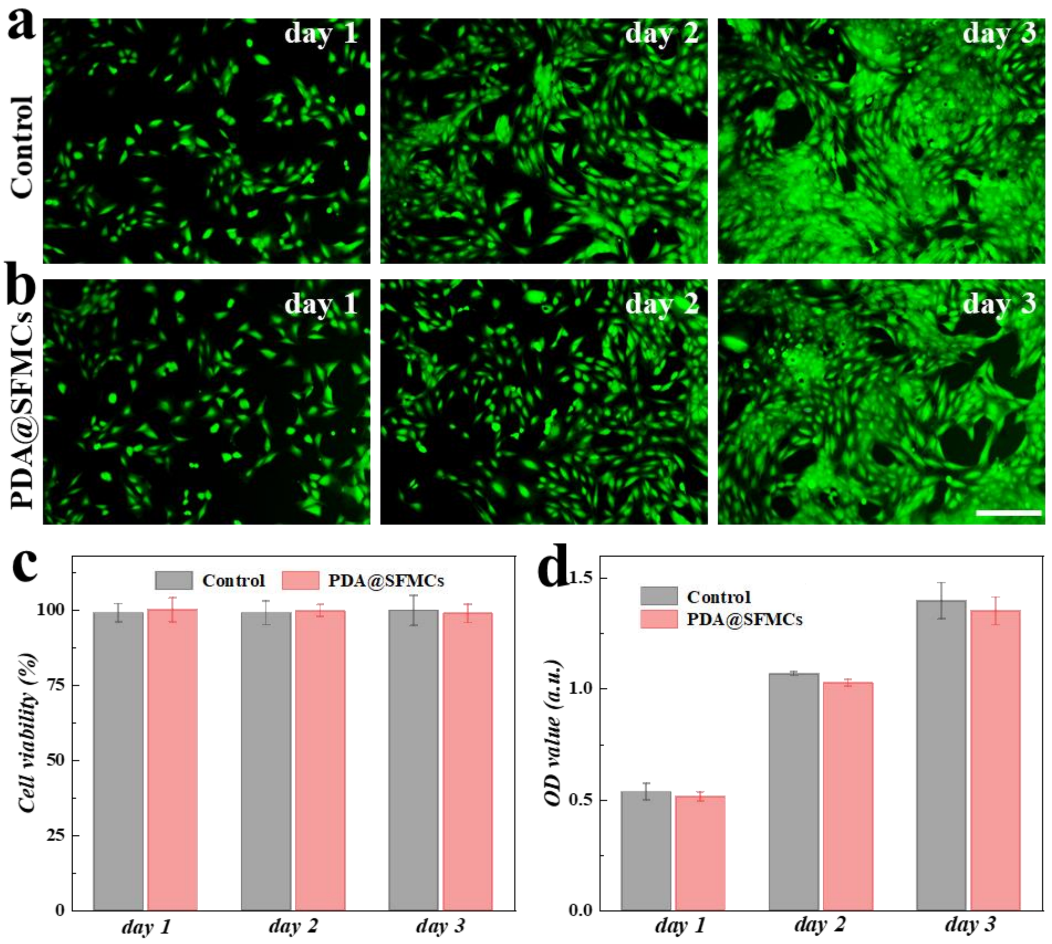

Figure S3. Biocompatibility test of resultant PDA@SFMCs. (a) Live/dead staining images of HEI-OC1 cells at days 1, 2, 3 of the control group. (b) Live/dead staining images of HEI-OC1 cells cocultured with PDA@SFMCs at days 1, 2, 3. (c) Statistic of live/dead staining images. (d) CCK-8 detection. Scale bar is 200 μm.

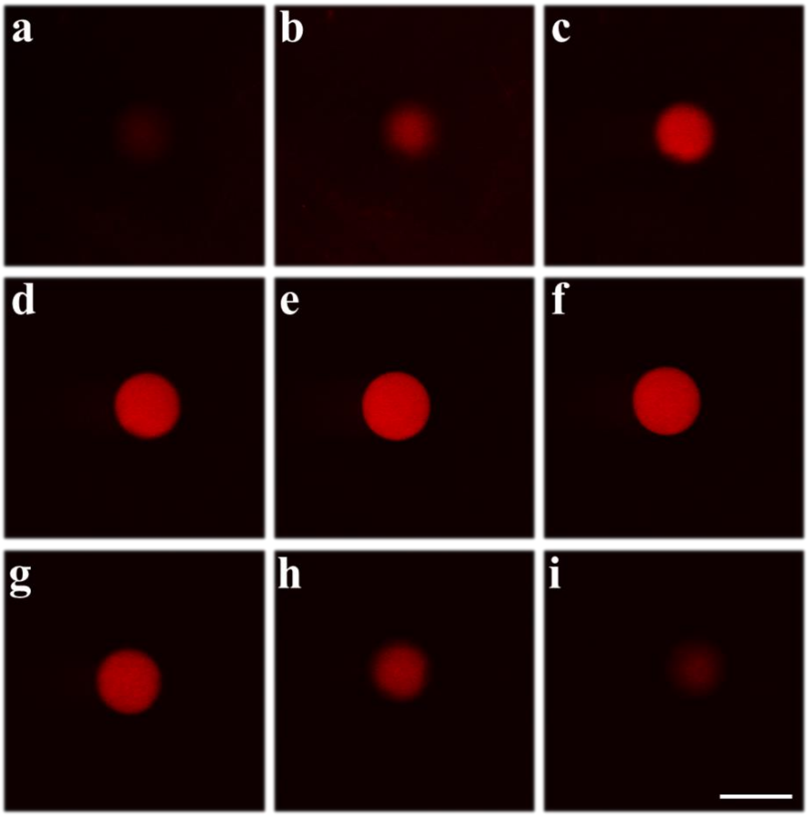

Figure S4. Layer-by-layer laser scanning photographs of RHB-loaded PDA@SFMCs. t=12 h. Scale bar is 50 μm.

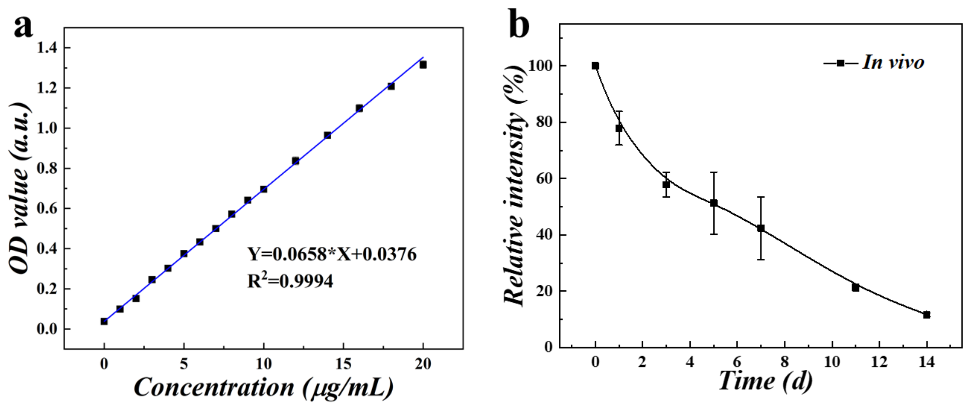

Figure S5. (a) Standard curve of RHB in APL. (b) Statistical analysis of IVIS images in Figure 3d.

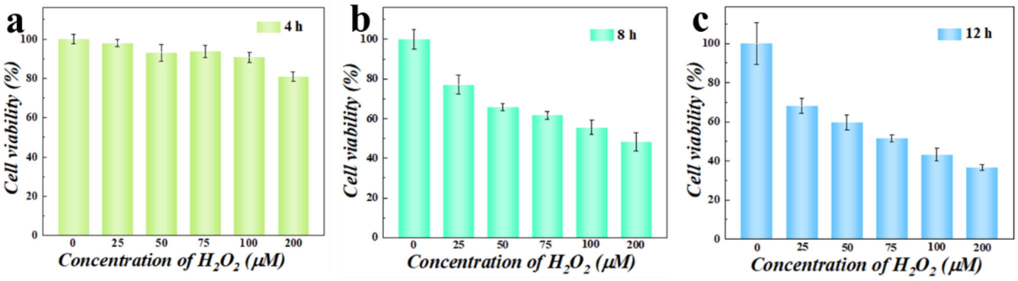

Figure S6. Relative cell viability of HEI-OC1 cells after exposure to H_2O_2 at various concentrations and with different treating periods.

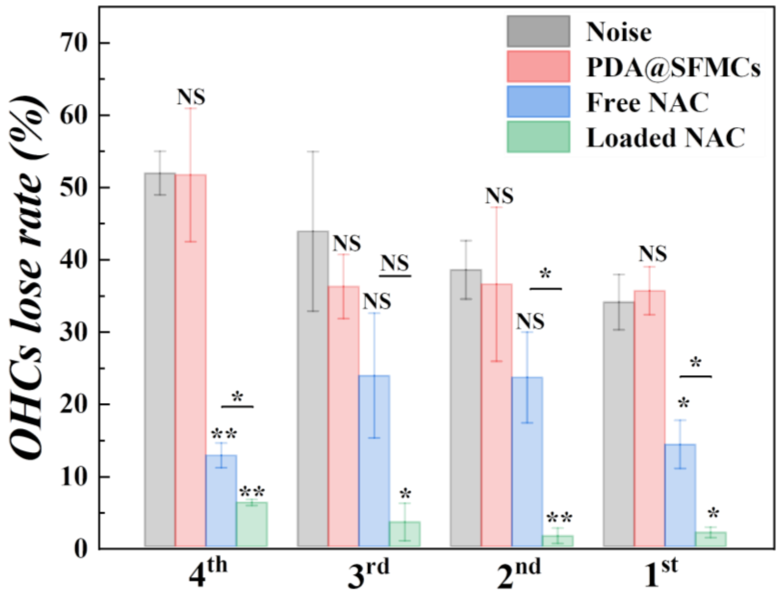

Figure S7. Statistical analysis of the OHCs at each turn in different groups.
